# Olive leaf extract supplementation improves the vascular and metabolic alterations associated with aging in Wistar rats

**DOI:** 10.1038/s41598-021-87628-7

**Published:** 2021-04-14

**Authors:** Daniel González-Hedström, Ángel Luís García-Villalón, Sara Amor, María de la Fuente-Fernández, Paula Almodóvar, Marin Prodanov, Teresa Priego, Ana Isabel Martín, Antonio Manuel Inarejos-García, Miriam Granado

**Affiliations:** 1 Departamento de Fisiología, Facultad de Medicina, Universidad Autónoma de Madrid, Madrid, Spain; 2 Pharmactive Biotech Products S.L. Parque Científico de Madrid, Avenida del Doctor Severo Ochoa, 37 Local 4J, Alcobendas, 28108 Madrid, Spain; 3 Departamento de Química Física Aplicada, Facultad de Ciencias, CIAL (CEI, CSIC-UAM), Universidad Autónoma de Madrid, Madrid, Spain; 4 Departamento de Fisiología, Facultad de Medicina, Universidad Complutense de Madrid, Madrid, Spain; 5 Instituto de Salud Carlos III, CIBER Fisiopatología de La Obesidad Y Nutrición, Madrid, Spain

**Keywords:** Plant sciences, Cardiology, Diseases, Endocrinology

## Abstract

Olive leaves are rich in bioactive substances which exert anti-inflammatory, antioxidant, insulin-sensitizing and antihypertensive effects. The aim of this study was to analyze the possible beneficial effects of an olive leaf extract (OLE) rich in secoiridoids and phenolic compounds on the aging-induced metabolic and vascular alterations. Three experimental groups of rats were used: 3-month-old rats, 24-month-old rats and 24-month-old rats supplemented 21 days with OLE (100 mg/kg). Administration of OLE to aged rats decreased the weight of adrenal glands and prevented the aging-induced loss of body weight and muscle mass. In the serum, OLE reduced the circulating levels of LDL-cholesterol and IL-6 and increased the concentrations of leptin and adiponectin. In the liver OLE attenuated the decreased gene expression of SOD-1, GSR, GCK and GSK-3β and reduced the aging-induced overexpression of NOX-4, Alox-5, iNOS and TNF-α. In aorta segments, OLE prevented endothelial dysfunction and vascular insulin resistance and improved vasoconstriction in response to KCl and NA. Improvement in vascular function was associated with the attenuation of the alterations in the gene expression of COX-2, IL-6, GPx, NOX-1 and IL-10. In conclusion, OLE exerts anti-inflammatory and antioxidant effects in aged rats and attenuates the alterations in vascular function associated with aging.

## Introduction

Aging is related with structural and functional alterations of vital organs. This is associated with the loss of physiological integrity, resulting in a progressive decline of homeostasis and a reduced capacity to respond to environmental stimuli, which contributes to the apparition of age-related co-morbidities^1^. Aging-associated co-morbidities include neurological, respiratory, oncologic, immune and/or metabolic disorders^2^. Among the latter, metabolic syndrome, a condition that is characterized by the co-existence of abdominal obesity, hypertriglyceridemia, fasting hyperglycemia, decreased HDL serum concentrations and hypertension, is one of the most prevalent among aged individuals^3^. Moreover, aging is considered the major risk factor for the development of cardiovascular diseases, which represent the leading cause of death in developed countries^4^. Aging-related cardiovascular alterations are the result, at least in part, of increased vasoconstriction and reduced endothelium-induced vasodilation which derives in enhanced incidence of arterial hypertension and atherosclerosis^5^.

In the last decades, the progressive increase in the number of elderly persons has represented a major sanitary and economic problem worldwide, due to the increment of age-related diseases. Furthermore, the proportion of the world’s population over 65 years is expected to double in the next decades, with this fact producing an increased incidence of age-related diseases and therefore a huge burden on healthcare systems^6^. For this reason, there is a strong interest in the development of new procedures and strategies that may slow the apparition of these disturbances.

Mediterranean diet, which includes the consumption of olive-derived products, is reported to exert several beneficial effects in old patients^7,8^ and for this reason, is recommended in the dietary guidelines for a healthy lifestyle^9^. In addition to olive oil, leaves from olive tree have been also used in traditional herbal medicine, and recent studies demonstrate a positive effect of olive leaf extracts (OLE) improving the alterations associated with the metabolic syndrome, such as dyslipidemia and insulin resistance^10,11^. These beneficial effects are attributed to the phenolic compounds present in the olive leaves that have potent antioxidant effects^12^. Indeed, OLE have been shown to exert antioxidant effects on culture cells^13^, invertebrates^14^ and mammals^15,16^.

In addition, since OLE reduces inflammation in several tissues and organs including vascular endothelial cells^17^, it may also be effective for the treatment/and or prevention of the age-related cardiovascular impairment. In this regard, there are some studies reporting the beneficial effects of OLE improving arterial hypertension both in rodents^18,19^ and in humans^20,21^, although the exact mechanisms of this antihypertensive effects are not well known.

Even though there is ample evidence about the beneficial effects of OLE on dyslipidemia and insulin resistance in metabolic syndrome^10,22^, its effects on insulin sensitivity and vascular function in the context of aging has not been studied yet. Thus, the aim of this study was to analyze the possible beneficial effects of a new OLE on the cardiometabolic alterations associated with aging in rats, and the possible antioxidant and/or anti-inflammatory mechanisms that may underlie this protection.

## Results

### Chemical characterization of OLE

According to the method described by Talhaoui et al.^23^ total flavonoid concentration was over 1.0% (1.15 ± 0.06%, dry basis).

The used RP-HPLC-PAD-MS analytical methodology allowed the identification of 32 constituents of the studied olive-tree leaf extract (Table 1). Eleven of them, protocatechuic acid, hydroxytyrosol, tyrosol, oleuropein, verbascoside, oleacein, quercetin-3-*O*-rutinoside, quercetin-3-*O*-glucoside, apigenin-7-*O*-rutinoside, luteolin and apigenin were identified unambiguously by co-elution and comparison with the retention time and the mass and UV spectra of commercial reference substances. The rest of the peaks were identified taking into account their chromatographic (retention time and order of elution), spectroscopic (ultraviolet–visible (UV–VIS) spectra), and spectrometric (molecular and typical fragment ions) characteristics.

**Table 1:** Identification of phenolic compounds and flavonoids by HPLC–MS in the OLE. For each compound, the ultraviolet–visible absorption spectrum (UV–VIS), [M-H]^−^ and fragment ions are shown.

T_R_ (min)	Molecule	UV–VIS (nm)	[M-H]^−^ (m/z)	Product ions (m/z)
14.1	3,4-DHBA (protocatechuic acid)	225/260/294	153.0	
14.8	Loganic accid	230	375.0	213.0
15.6	3,4-DHPE (hydroxytyrosol)	227/280	153.1 (305.2)*	123.2
18.0	3,4-DHPE-glucoside	228/278	315.0	153.1
20.6	DM-EA glucoside (isomer 1)	233	389.1 (779.2)*	227.0, 183.0
34.0	DM-EA glucoside (isomer 2)	236	389.2	345.1
37.9	EA glucoside (isomer 1)	240	403.0	241.1, 139.1
40.6	EA glucoside (isomer 2)	240	403.0 (807.3)*	100.9
50.4	EA glucoside (isomer 3)	238	403.1 (807.1)*	
56.6	EA glucoside (isomer 4) + hydroxyloganin	246/247/323	403.1 405.1 (811.0)*	240.9
62.7	EA (isomer 1)	239	241.0	165.1, 139.1, 127.1, 121.1, 111.2, 101.0
64.5	quercetin-3-O-rutinoside	258/304sh/358	609.0	301.1
66.0	quercetin-3-O-glucoside	242/299sh/333	436.0	301.0
66.7	EA (isomer 2)	240	241.0	
70.2	Verbascoside + luteolin-O-hexoside	239/288sh/334 228/267sh/341	623.1 447.1 (895.2)*	477.0
72.6	3,4-DHPE-DHEA-glucoside + 3,4-DHPE-DA-EDA (oleacein)	230/277	543.0 (1087.4)* 319.0	513.0 275.1, 241.1, 139.2
78.1	Apigenin-7-O-rutinoside	257/333	577.0	
85.2	Diosmin + luteolin-O-hexoside	270/332	607.0 447.1	461.1 285.0
86.7	3,4-DHPE-EA-glucoside (oleuropein)	280	539.1	377.0
91.8	3,4-DHPE-EA-glucoside (oleuropein,isomer 2)	282	539.2	403.1, 377.0
95.9	3,4-DHPE-EA-glucoside (oleuropein,isomer 3)	281	539.9	403.1, 377.1, 345.0, 307.0, 275.0
97.0	Luteolin	227/267/350	285.0	
98.9	4-HPE-EA-glucoside (ligustroside)	281	523.2	509.0, 361.0
112.9	Apigenin	232/287sh/314	269.0	
115.6	3,4-DHPE-EA (isomer 1)	281	377.1	307.1, 241.0
118.1	3,4-DHPE-EA (isomer 2)	282	377.0	345.1, 307.0, 275.0, 149.1, 139.1
119.9	3,4-DHPE-EA (isomer 3)	282	377.1	275.1, 240.9, 139.1
122.6	3,4-DHPE-EA (isomer 4)	281	377.1	307.0, 275.0, 241.2, 138.9

3,4-DHBA = 3,4-dihydroxybenzoic acid; 3,4-DHPE = 3,4-dihydroxyphenylethanol (hydroxytyrosol); 4-HPE = 4-hydroxyphenylethanol (tyrosol); DM = demethyl; EA = elenolic acid; DHEA = dihydro elenolic acid; DA-EDA = deacetoxy elenolic acid dialdehyde form; * double ion.

The results in Table 1 showed the presence of a wide diversity of secondary olive leaf metabolites that include secoiridoids, simple and flavonoid phenolic compounds and iridoids. Among secoiridoids, four elenolic acid (EA) and two demethyl (DM) elenolic acid glucosides were identified, together with two isomers of elenolic acid. Regarding simple phenolic compounds, the most diverse was the group of phenylethanoids, mainly represented by hydroxytyrosol and its four isomer esters with elenolic acid, one ester with deacetoxy elenolic acid dialdehyde form (oleacein), three isomer esters with elenolic acid glucoside (oleuropein), one ester with dihydro elenolic acid glucoside, one glucoside and one ester of tyrosol with elenolic acid glucoside (ligustroside). This group includes also the hydroxytyrosol derivative verbascoside and the dihydroxybenzoic acid, protocatechuic acid. Flavonoids were composed by the two flavonols quercetin-3-*O*-rutinoside and quercetin-3-*O*-glucoside, and six flavones, luteolin, two luteolin hexoside isomers, diosmin, apigenin and apigenin-7-*O*-rutinoside. Two iridoids were detected also, loganic acid and hydroxyloganin.

### Body weight and food and water intake changes induced by aging and OLE treatment

**Figure 1:**
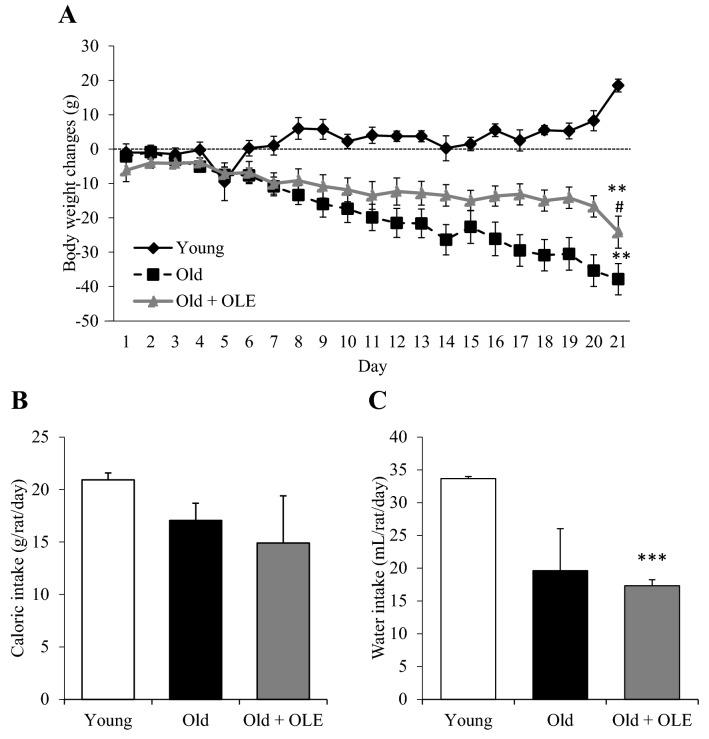
Effects of aging and a 21-day treatment with the OLE on rat body weight gain (**A**), daily food (**B**) and water (**C**) intake. Values are represented as mean ± SEM. **p < 0.01 vs. Young; ^#^*p* < 0.05 vs. Old.

Body weight gain is shown in Fig. 1A. The two-way ANOVA revealed significant differences among experimental groups for both factors (Time: *p* < 0.001 and experimental group: *p* < 0.001). The post-hoc analysis showed that whereas the young rats gained weight over the three-week treatment, the old rats significantly lost weight (*p* < 0.01). This body weight loss was attenuated in aged rats treated with OLE (*p* < 0.01). Daily water intake was significantly decreased in old rats treated with OLE compared to young animals (*p* < 0.001) (Fig. 1C). However, caloric intake was unchanged between groups (Fig. 1B).

### Effects of aging and OLE on organ weights

Absolute and relative organ weights from young and old rats treated with vehicle or with OLE are shown in Table 2. Compared to young animals, old rats showed increased absolute weights of heart (*p* < 0.01, liver (*p* < 0.05), epidydimal visceral (*p* < 0.001), white lumbar subcutaneous (*p* < 0.01), interscapular brown (*p* < 0.05) and periaortic (*p* < 0.05) adipose tissues. Treatment with OLE prevented the aging-induced decrease in the absolute weight of gastrocnemius muscle (*p* < 0.05). Relative organ weights followed the same pattern than absolute weights except for the heart, and the interscapular and periaortic adipose tissue depots that were not significantly increased in old untreated rats compared to young rats. OLE treatment increased the relative weight of the interscapular brown adipose tissue compared to untreated old rats (*p* < 0.05).

**Table 2:** Organ weights of young rats, old rats and old rats treated with olive leaves extract (OLE).

	Young	Old	Old + OLE
Heart (mg)	1,503.8 ± 78.8	1,891.3 ± 55.6**	1,845.8 ± 78.9**
Heart (mg/100 g body weight)	314.6 ± 10.8	291.0 ± 14.4	264.6 ± 13.7*
Epidydimal visceral adipose tissue (mg)	9,843.1 ± 1075.0	20,475.4 ± 1451.5***	21,545.9 ± 1908.0***
Epidydimal visceral adipose tissue (mg/100 g body weight)	2,107.1 ± 183.7	3,201.8 ± 139.6***	2,970.7 ± 265.1**
Lumbar subcutaneous adipose tissue (mg)	4,992.4 ± 787.5	27,905.7 ± 6192.6***	28,619.6 ± 2906.6***
Lumbar subcutaneous adipose tissue (mg/100 g body weight)	1,063.1 ± 139.6	3,974.2 ± 661.4***	4,249.9 ± 396.9***
Interscapular brown adipose tissue (mg)	496.3 ± 50.0	810.8 ± 119.4*	1011.8 ± 154.6***
Interscapular brown adipose tissue (mg/100 g body weight)	105.6 ± 9.8	111.1 ± 12.9	140.4 ± 14.4*^#^
Periaortic adipose tissue (mg)	180.0 ± 26.0	307.9 ± 47.7**	397.5 ± 57.8***
Periaortic adipose tissue (mg/100 g body weight)	39.3 ± 5.9	47.8 ± 7.5	55.2 ± 5.9*
Liver (mg)	13,465.0 ± 425.6	14,886.9 ± 566.9*	15,733.9 ± 588.5**
Liver (mg/100 g body weight)	2,932.8 ± 103.6	2,204.2 ± 138.6***	2,343.0 ± 92.6**
Soleus (mg)	180.8 ± 12.6	163.4 ± 10.8	173.1 ± 11.6
Soleus (mg/100 g body weight)	39.2 ± 2.1	26.0 ± 1.6***	24.8 ± 2.0***
Gastrocnemius (mg)	2,473.4 ± 50.7	2,239.1 ± 101.9*	2,560.9 ± 123.0^#^
Gastrocnemius (mg/100 g body weight)	534.4 ± 12.1	329.4 ± 15.7***	375.5 ± 11.1***^#^

Data are represented as mean value ± SEM; n = 6–11 samples/group.

*p < 0.05 vs. Young; **p < 0.01 vs. Young; ***p < 0.001 vs. Young; ^#^*p* < 0.05 vs. Old.

### Effects of aging and OLE on lipid profile, serum levels of metabolic hormones and HOMA-IR index

Aging was associated with increased circulating levels of total lipids (*p* < 0.05), triglycerides (*p* < 0.05), total cholesterol (*p* < 0.05), LDL-cholesterol (*p* < 0.01), insulin (*p* < 0.01), leptin (*p* < 0.01), adiponectin (*p* < 0.001) and HOMA-Index (*p* < 0.05). Treatment with OLE prevented the aging-induced increase in LDL-cholesterol and further increased the serum levels of leptin and adiponectin (*p* < 0.05 for both) (Table 3).

**Table 3:** Lipid profile, hormone concentrations and inflammatory parameters in the serum of young rats, old rats and old rats treated with olive leaves extract (OLE).

	Young	Old	Old + OLE
Glycemia (mg/dL)	90.7 ± 3.8	72.4 ± 11.1	62.7 ± 10.7*
Total Lipids (mg/dL)	853 ± 63	1051 ± 37*	1173 ± 87**
Triglycerides (mg/dL)	97.6 ± 13.5	158.5 ± 36.4*	169.5 ± 11.7**
Total Cholesterol (mg/dL)	135.1 ± 15.3	199.3 ± 13.9*	194.0 ± 26.3
LDL-cholesterol (mg/dL)	28.8 ± 2.7	47.8 ± 2.6**	33.6 ± 1.4^###^
HDL-cholesterol (mg/dL)	15.7 ± 0.6	15.7 ± 2.7	15.8 ± 0.6
Insulin (ng/mL)	17.7 ± 5.2	81.3 ± 32.2**	30.0 ± 3.4
HOMA-Index	1.75 ± 0.3	13.1 ± 5.4*	5.5 ± 2.3*
Leptin (ng/mL)	11.82 ± 1.4	30.2 ± 5.9**	53.0 ± 7.7***^#^
Adiponectin (mg/dL)	67.1 ± 6.7	108.9 ± 4.3***	123.5 ± 5.5***^#^
Interleukin-6 (pg/mL)	135.6 ± 7.1	188.7 ± 24.1*	146.7 ± 7.9^#^
TNFα (pg/mL)	0.1 ± 0.1	1.8 ± 0.9*	0.82 ± 0.6

Data are represented as mean value ± SEM; n = 6–11 samples/group.

*p < 0.05 vs. Young; **p < 0.01 vs. Young; ***p < 0.001 vs. Young; ^#^*p* < 0.05 vs. Old; ^###^*p* < 0.001 vs. Old.

### Effects of aging and OLE on serum inflammatory parameters

Old rats showed higher serum levels of the inflammatory markers IL-6 and TNFα compared to young rats (*p* < 0.05 for both) and treatment with the OLE prevented the aging-induced increase in IL-6 (*p* < 0.05) (Table 3).

### Effects of aging and OLE on gene expression of insulin sensitivity-related enzymes in the liver

**Figure 2:**
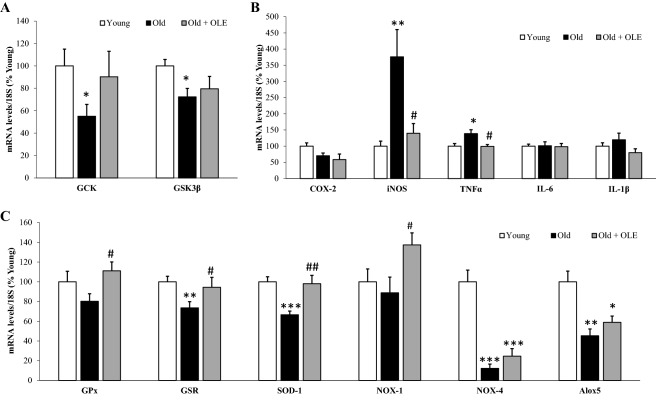
Effects of aging and a 21-day treatment with the OLE on mRNA concentrations of Glucokinase and Glycogen synthase kinase 3β (**A**), ciclooxigenase-2, inducible Nitric Oxide Synthase, Tumor Necrosis Factor α, Interleukin 6 and Interleukin 1β (**B**) and Glutathione Peroxidase, Glutathione Reductase, Super Oxide Dismutase 1, NADPH oxidase 1 and 4, and Lipoxygenase (**C**) in rat livers. Values are represented as mean ± SEM. *p < 0.05 vs. Young; **p < 0.01 vs. Young; ***p < 0.001 vs. Young; ^#^*p* < 0.05 vs. Old; ^##^*p* < 0.01 vs. Old.

The mRNA levels of the insulin sensitivity-related enzymes GCK and GSK3β were significantly downregulated in untreated old rats compared to young animals (Fig. 2A; *p* < 0.05 for both) but not in aged rats treated with OLE.

### Effects of aging and OLE on gene expression of inflammatory and oxidative stress markers in the liver

The hepatic overexpression of the pro-inflammatory markers iNOS (*p* < 0.01) and TNFα (*p* < 0.05) associated with aging was prevented by the treatment with OLE (*p* < 0.05 for both) (Fig. 2B).

Old rats also showed reduced gene expression in the liver of the antioxidant enzymes GSR and SOD-1 (*p* < 0.05 and *p* < 0.001, respectively), as well as NOX-4 (*p* < 0.001) and Alox5 (*p* < 0.01). The treatment of old rats with OLE did not affect the gene expression of NOX-4 and Alox5, but it prevented both the aging-induced reduction of GSR (*p* < 0.05) and SOD-1 (*p* < 0.01) and significantly upregulated the mRNA levels of GPx (*p* < 0.05) and NOX-1 (*p* < 0.05) (Fig. 2).

### Effects of aging and OLE on gene expression of inflammatory and oxidative stress markers in cardiac tissue

**Figure 3:**
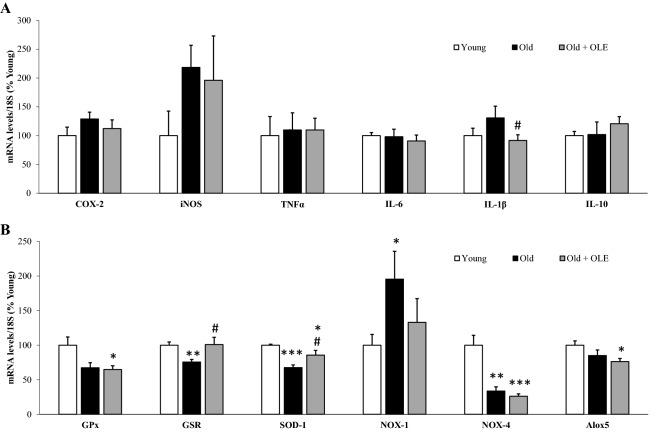
Effects of aging and a 21-day treatment with the OLE on mRNA concentrations of ciclooxigenase-2, inducible Nitric Oxide Synthase, Tumor Necrosis Factor α, Interleukin 6, Interleukin 1β and Interleukin 10 (**A**) and Glutathione Peroxidase, Glutathione Reductase, Super Oxide Dismutase 1, NADPH oxidase 1 and 4, and Lipoxygenase (**B**) in rat hearts. Values are represented as mean ± SEM.* *p* < 0.05 vs. Young; **p < 0.01 vs. Young; ***p < 0.001 vs. Young; ^#^*p* < 0.05 vs. Old.

There were no significant changes in the mRNA levels of inflammatory markers in the cardiac tissue of old rats compared to young ones. However, supplementation with OLE to old rats significantly downregulated the gene expression of IL-1β (*p* < 0.05) compared to untreated ones (Fig. 3A).

The gene expression of the antioxidant enzymes GSR and SOD-1 was reduced in old compared to young rats (*p* < 0.01 and *p* < 0.001, respectively) whereas the mRNA levels of the pro-oxidant enzyme NOX-1 were significantly increased (*p* < 0.05). Treatment with OLE prevented the aging-induced reduction in GSR and SOD-1 mRNA levels (*p* < 0.05 for both). The gene expression of NOX-4 was reduced in old rats regardless of whether they had been treated (*p* < 0.001) or not (*p* < 0.01) with OLE (Fig. 3B).

### Aortic vasoconstriction changes induced by aging and OLE treatment

**Figure 4:**
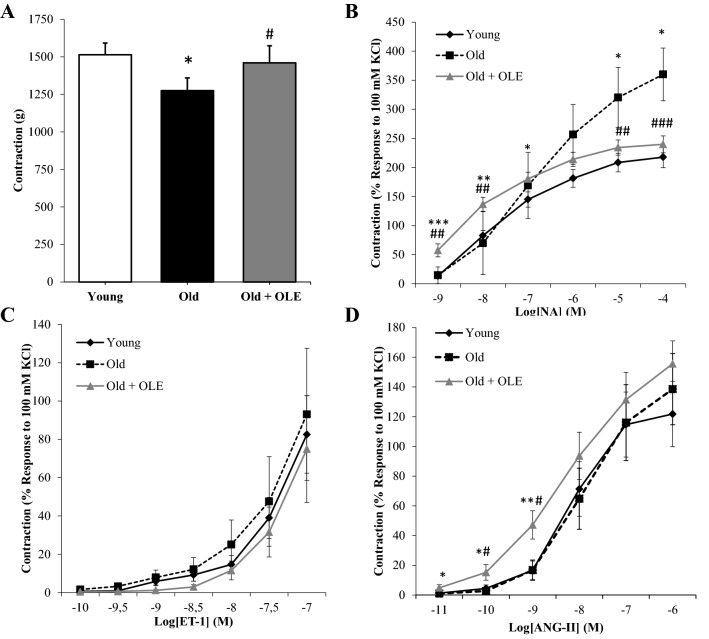
Effects of aging and a 21-day treatment with the OLE on the contraction response of abdominal aortic segments to potassium chloride 100 mM (**A**), norepinephrine (**B**), endothelin-1 (**C**) and to angiotensin-II (**D**). Values are represented as mean ± SEM. *p < 0.05 vs. Young; **p < 0.01 vs. Young; ***p < 0.001 vs. Young; ^#^*p* < 0.05 vs. Old; ^##^*p* < 0.01 vs. Old; ^###^*p* < 0.001 vs. Old.

**Figure 5:**
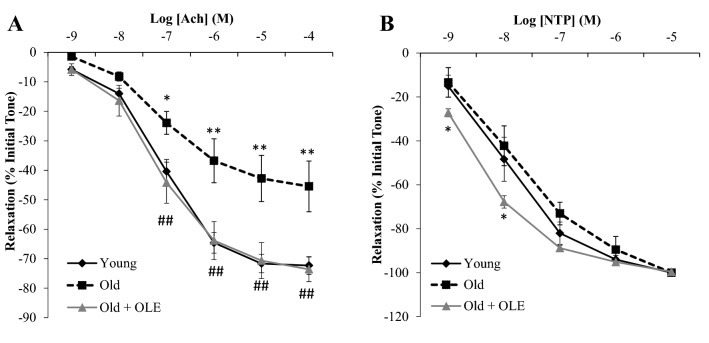
Effects of aging and a 21-day treatment with the OLE on the relaxation response of thoracic aortic segments to acetylcholine (**A**) and to sodium nitroprusside (**B**). Values are represented as mean ± SEM. *p < 0.05 vs. Young; **p < 0.01 vs. Young; ^##^*p* < 0.01 vs. Old.

Arterial vasoconstriction of abdominal aorta segments to KCl (A), Noradrenaline (B), Endothelin-1 (C) and Angiotensin II (D) is shown in Fig. 4. Aging did not modify arterial vasoconstriction in response to either ET-1 (Fig. 4C) or Ang-II (Fig. 4D). However, aorta segments from old untreated rats showed a decreased vasoconstrictor response to KCl (*p* < 0.05; Fig. 4A), and an increased vascular contraction in response to accumulative concentrations of NA (*p* < 0.01; Fig. 5B).

Treatment with OLE prevented the aging-induced decrease in arterial vasoconstriction in response to KCl (*p* < 0.05) and the aging-induced increased vasoconstriction in response to NA (*p* < 0.01). Nonetheless, the contraction to AngII was significantly increased at low doses by treatment with the OLE (*p* < 0.05; Fig. 4D).

### Changes on endothelium-dependent and independent relaxation in aorta segments induced by aging and OLE treatment

The endothelium-dependent relaxation in response to acetylcholine was significantly reduced in aorta segments from old rats (*p* < 0.01; Fig. 5A) and this effect was totally reverted by OLE treatment (*p* < 0.01; Fig. 5A). On the other hand, the endothelium-independent relaxation in response to sodium nitroprusside (NTP) was not modified in untreated old rats, whereas OLE treatment significantly increased endothelium-independent relaxation at low concentrations of NTP (*p* < 0.05; Fig. 5B).

#### Aortic insulin vascular relaxation and activation of PI3K/Akt pathway

**Figure 6:**
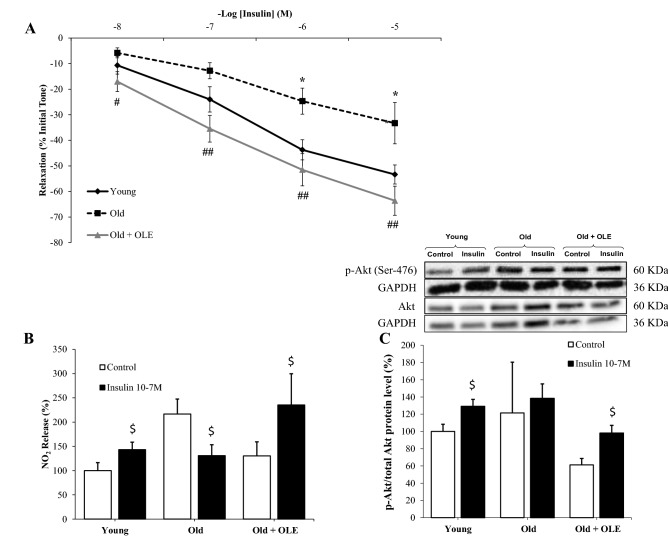
Effects of aging and a 21-day treatment with the OLE on the relaxation response of thoracic aortic segments to insulin (**A**), on the nitrites release from aorta segments to culture medium in presence/absence of insulin (10^−7^ M) (**B**), and on the ratio between protein levels of p-Akt and Akt in aorta (**c**). Values are represented as mean ± SEM. *p < 0.05 vs. Young; ^#^*p* < 0.05 vs. Old; ^##^*p* < 0.01 vs. Old; ^$^*p* < 0.05 vs. Control.

Despite insulin induced dose‐dependent relaxation of aorta segments in all experimental groups (Fig. 6A), aging was related to a reduced relaxation response compared to young rats (*p* < 0.05) and significantly improved when supplemented with OLE (*p* < 0.01).

Incubation of aorta segments with insulin increased the release of nitrites to the culture medium and the ratio p-Akt/Akt in arterial tissue from young (*p* < 0.05) and old rats treated with the OLE (*p* < 0.05), but not in untreated old rats (Fig. 6B and C).

#### Effects of aging and OLE on gene expression of inflammatory and oxidative stress markers in the aorta

**Figure 7:**
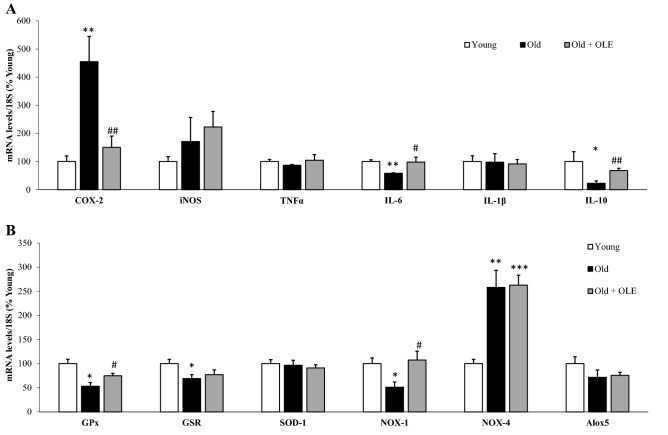
Effects of aging and a 21-day treatment with the OLE on mRNA concentrations of ciclooxigenase-2, inducible Nitric Oxide Synthase, Tumor Necrosis Factor α, Interleukin 6, Interleukin 1β and Interleukin 10 (**A**) and Glutathione Peroxidase, Glutathione Reductase, Super Oxide Dismutase 1, NADPH oxidase 1 and 4, and Lipoxygenase (**B**) in rat aortas. Values are represented as mean ± SEM. *p < 0.05 vs. Young; **p < 0.01 vs. Young; ***p < 0.001 vs. Young; ^#^*p* < 0.05 vs. Old; ^##^*p* < 0.05 vs. Old.

Untreated old rats showed a significant increase in the aortic gene expression of COX-2 (*p* < 0.01) and NOX-4 (*p* < 0.001) and a significant reduction in the mRNA levels of IL-6 (*p* < 0.01), IL-10 (*p* < 0.05), GPx (*p* < 0.05), GSR (*p* < 0.05) and NOX-1 (*p* < 0.05). The treatment with OLE attenuated the aging-induced changes in COX-2 (*p* < 0.01), IL-6 (*p* < 0.05), GPx (*p* < 0.05), NOX-1 (*p* < 0.05) and IL-10 (*p* < 0.01) mRNA levels, but not in NOX-4 (Fig. 7).

## Discussion

This study provides evidence about the effects of a new OLE rich in flavonoids and secoiridoids alleviating the cardiometabolic disorders associated with aging. There are previous studies reporting the positive effects of treatments with olive leaf extracts decreasing oxidative stress in major organs of aged rats^15^ and reducing aging-induced neurodegeneration in humans^24^ and in rodents^25^. However, to our knowledge, this is the first study that demonstrates the beneficial metabolic and cardiovascular effects of an olive leaf extract in aged animals. Importantly, our results showed that the OLE used in this study exerts its cardiometabolic protective effects at a considerably lower dose than the extract used by other authors (100 mg/kg vs 500 and 1000 mg/kg)^15^ and in a shorter period of time (three weeks vs 6 months)^25^. The dose used in this study was selected based in previous studies found in the literature in which the same dose of OLE had significant effects in rats preventing fluoxetine-induced hepatotoxicity through attenuation of oxidative stress, inflammation, and apoptosis^26^. Likewise, a very recent study shows how the same dose of OLE prevents type-2 diabetes in rats and decreases the expression of liver superoxide dismutase as well as the total antioxidant capacity in the plasma^27^.

Aged rats showed increased adiposity, increased plasma levels of leptin, total and LDL-cholesterol and triglycerides and decreased muscle mass. It is reported that, in addition to adiposity and dyslipidemia^28^, this loss of muscle mass, so called sarcopenia, is a common characteristic among individuals suffering from metabolic syndrome^29^. In addition, several parameters related to insulin sensitivity were also impaired with aging, like elevated insulin plasma levels and HOMA index, and decreased gene expression of GCK and GSK3β in the liver. These alterations were in agreement with some of the characteristics of metabolic syndrome in elderly patients^3^.

OLE supplementation in the drinking water for three weeks to old rats partially prevented age-induced weight loss over the three-week treatment. Since there were no significant changes in cumulative intake among the three experimental groups, this effect was not attributable to modifications in caloric intake. The effect of OLE attenuating body weight loss in old rats may be explained, at least in part, by the significant increase in muscle mass in old treated rats since skeletal muscle accounts for approximately 40% of total body mass. In agreement with this, it is reported that supplementation with secoiridoids from olive oil attenuates sarcopenia in overweight and obese older adults^30^.

It is also reported that aging is associated with a chronic state of low-grade inflammation, with this fact being involved in the development of aging-associated comorbidities^31^, and particularly in the mechanisms of the metabolic impairment in this condition^32^. In agreement with this, untreated old rats exhibited elevated circulating levels of IL-6 and TNFα and these concentrations were reduced by treatment with the OLE in the case of IL-6. Likewise, other studies have demonstrated the systemic anti-inflammatory capacity of different extracts of olive leaves both in humans^20,33,34^ and in rodents^35,36^. This effect is most likely due to the presence of phenolic compounds in the extract since it is reported in several in vivo and in vitro models that the flavonoids and phenolic secoiridoids identified in the OLE used in this study, such as luteolin and its derivatives, verbascoside, oleacein and most of the identified hydroxytyrosol elenolates, exert powerful anti-inflammatory effects, decreasing both the systemic and tissue levels of IL-6^37–39^. The OLE-induced decrease in the circulating levels of IL-6 may be related, at least in part, with its beneficial effects attenuating muscle loss, since a positive relationship between serum IL-6 and sarcopenia has been reported^40^. Moreover, OLE treatment also prevented the aging-induced upregulation of IL-6 mRNA levels in gastrocnemius muscle (Young: 100 ± 8; Old: 152 ± 12**; Old + OLE: 119 ± 11^#^), pointing to a local anti-inflammatory effect at the muscular level.

OLE treatment to old rats not only decreased the circulating levels of IL-6 but also improved dyslipidemia reducing the plasma levels of LDL-cholesterol. Consistent with our results, treatment with OLE is reported to improve the lipid profile both in rodents subjected to high fat diets^35,41–44^ and in a cohort of pre-hypertensive men^20^. However, OLE treatment increased circulating leptin and adiponectin levels in aged rats, which may be related with the slightly higher adiposity of these rats compared to the untreated ones. Although the increase in leptin levels in individuals with increased adiposity, both humans and experiments animals, is clearly demonstrated^45^ , the association of circulating adiponectin levels with obesity and metabolic syndrome is controversial. In this regard, it is extensively reported that adiponectin levels are decreased in obese individuals^46,47^ and in experimental models of genetic obesity^48^. However, it is not that clear in experimental models of diet-induced obesity in which both decreased^48^ and increased^49^ circulating levels of this adipokine have been described. Indeed, increased adiponectin serum levels have been found increased overtime in a study that examined prospectively the course of circulating adiponectin levels during development of metabolic alterations in a diet-induced rat model of metabolic syndrome^50^. Likewise, in experimental models of aging both unchanged^51^ and increased adiponectin serum concentrations have been found^52^. Thus, it is possible that the commonly decreased adiponectin serum levels observed in humans in a context of obesity and/or metabolic syndrome are not necessarily reproduced in experimental models of metabolic syndrome induced by either aging or diet.

Glycemia and hepatic pAkt/Akt ratio (Supplementary data; Fig. 3A) were unchanged between young and old rats fed ad libitum but they were significantly decreased in aged rats treated with OLE. These results are in agreement with other studies in which OLE is reported to exert antidiabetic effects both in rats^53,54^ and in humans^54^. Although the differences in HOMA index or insulin plasma levels between treated and untreated old rats were not statistically significant, our results showed evidence of preserved insulin sensitivity in the liver of treated animals. Likewise, other authors have reported increased insulin sensitivity in the liver of diabetic rats treated with OLE^55^. However, although in basal conditions the mRNA levels of GSK3β were significantly decreased in old untreated rats and not in old rats treated with OLE, the ratio pGSK3β/GSK3β was unchanged with aging and significantly increased in OLE treated animals (Supplementary data; Fig. 3B). Thus, further studies are required to dilucidate the differences between hepatic GSK3β gene and protein expression, not only in basal conditions but also in response to insulin stimulation.

Based on previous studies, the insulin sensitizing effects of OLE may be attributed to the presence of phenolic secoiridoids such as oleuropein and its products of hydrolysis, but also to other compounds such as hydroxytyrosol, tyrosol and ligustroside^10^. Since the development of insulin resistance and type 2 diabetes is related to increased inflammation and oxidative stress^56^, the increased insulin sensitivity in the liver may be related, at least in part, with the effect of OLE reducing the mRNA levels of the pro-inflammatory markers iNOS and TNF-α and increasing the gene expression of the antioxidant enzymes GPx, GSR and SOD-1. These results are in agreement with those described in other experimental models of liver damage, in which treatment with an OLE attenuates oxidative stress^26,57,58^, decreases the hepatic gene expression of proinflammatory cytokines^26,35,55,58,59^ and increases hepatic insulin sensitivity^55^. However, in heart and aorta tissues the gene expression of TNF-α was not affected neither by aging nor by OLE treatment. The OLE-induced reduction of TNF-α mRNA levels in the liver and not in other tissues could be explained, at least in part, because the liver, due to its key role in metabolic and detoxifying pathways, is probably more exposed to inflammatory stress and OLE may be effective only when there is inflammatory activation above basal levels. This may be related with the non-significant reduction of TNF-α circulating levels in OLE treated rats, since TNF-α plasma levels represent an average production in all organs and, for this reason, they show a higher variability.

Moreover, decreased hepatic insulin resistance in old rats treated with OLE may also be related to the increased circulating levels of the insulin sensitizing hormone adiponectin, as previously reported in adipose tissue of ovariectomized rats^60^ and obese mice treated with OLE^59^. Adiponectin has insulin-sensitizing and endothelium-protecting effects^61^, so these elevated levels may be related to the improved metabolic and vascular function in OLE treated rats. However, adiponectin levels are also correlated with incident falls^62^, which may be the result of a bone weakening effect of this hormone^63,64^ due to the stimulation of osteoclastogenesis^65^. Thus, further studies are required to determine OLE effects on bone density.

Our results show that aged rats not only suffered metabolic but also vascular alterations. The endothelium-dependent relaxation to acetylcholine was reduced in the aortas of untreated old rats indicating, as previously described^66,67^, that the ability of the endothelium to regulate the vascular tone in an aging context is altered. Likewise, the contraction to KCl was reduced indicating that smooth muscle function is also impaired^67^. However, the arterial contraction to norepinephrine was increased, suggesting that there is hypersensitivity of adrenergic receptors, as previously reported in an aging context both in humans and in rodents^66^. These vascular alterations were reversed by treatment with OLE, which normalized both the relaxation to acetylcholine and the contraction to KCl and norepinephrine. OLE also increased the endothelium-independent relaxation in response to NTP and the contraction to Ang II which were not altered by aging, suggesting that the action of OLE on the vascular system may be more extensive beyond counteracting the effects of aging. Other authors have reported beneficial effects of OLE in cardiovascular function reducing both cardiac output and total peripheral vascular resistance in spontaneously hypertensive rats^68^ and enhancing endothelium-dependent relaxation and eNOS activation through the reduction of oxidative stress^19^. In the present study OLE also improved the aging-induce endothelial dysfunction and reduced the adrenergic hypersensitivity in the aorta of old rats, which may also contribute to the antihypertensive effect. Paradoxically, a significant increase in the contraction of aorta segments to angiotensin II was also found. This finding may be explained by the effect of OLE reducing the angiotensin converting enzyme, as it has been described previously^69^, which may decrease the circulating levels of angiotensin II, thus resulting in compensatory hypersensitivity of angiotensin receptors.

As previously described^70^, our results also showed that aging is not only associated with insulin resistance in metabolic tissues, but also in the cardiovascular system where it decreases the vasodilation of aorta segments in response to insulin through an impaired activation of the PI3K/Akt pathway and decreased nitric oxide release. In a previous study, we reported that this impairment can be attenuated by a moderate protocol of caloric restriction^70^. Similarly, in this study we demonstrated that the treatment with OLE improved vasodilation of aorta segments in response to insulin and increased both the release of nitric oxide and the activation of the PI3K/Akt pathway in arterial tissue. To our knowledge this is the first study that reports a positive effect of OLE attenuating aging-induced insulin vascular resistance.

The protective effects of OLE on cardiovascular function may also be related to its anti-inflammatory and antioxidant effects, as treatment with OLE to old rats significantly reduced the gene expression of IL-1β in the heart and the mRNA levels of the inflammatory marker COX-2 in the aorta and increased the gene expression of the antioxidant enzyme GPx in the aorta and the expression of GSR and SOD-1 in the heart. Moreover, OLE treatment prevented the aging-induced downregulation of anti-inflammatory cytokine IL-10 in arterial tissue. Paradoxically an overexpression of the inflammatory marker IL-6 and the prooxidative enzyme NOX-1 in the aorta was also found. However, it has been proposed that in some cases nutritional phytochemicals may induce hormetic mechanisms which are based on activation of a moderate oxidative and/or inflammatory stress that activates compensatory mechanisms^71,72^.

Our results of gene expression of inflammatory and oxidative stress markers in cardiac and arterial tissue agree with previous studies that report the effects of both OLE and its components attenuating inflammation and DNA damage in human arterial endothelial cells^17^, preventing cardiac stiffness and fibrosis^44^ and reducing myocardial oxidative damage and atherosclerosis^73^.

In general, this study demonstrates that an OLE rich in flavonoids and secoiridoids alleviates the cardiometabolic disorders associated with aging. Further studies are required to assess if this treatment is also useful to prevent cardiometabolic alterations in aged humans as well as in experimental models of metabolic syndrome. Likewise, further studies are required to identify which of the specific compounds present in this extract are responsible for the protective cardiometabolic effects, although the functional effects of the OLE shown in this manuscript are probably not due to the contribution of individual molecules but to the overall composition of this natural extract.

## Conclusion

In conclusion, the results of the present study indicate that OLE is effective attenuating the metabolic and vascular alterations associated with aging possibly through the reduction of inflammation and oxidative stress. Therefore, it may constitute a useful strategy to improve cardiometabolic alterations in aged patients.

## Material and methods

All methods were carried out in accordance with relevant guidelines and regulations. Plant manipulations comply with national and international guidelines and legislation.

### Materials

Water soluble samples of olive leaf extract (OLE) from *Olea europaea* L. standardized in 30% of ortho-diphenols by UV/Vis were provided by Pharmactive Biotech Products S.L. (Madrid, Spain). All of them were in powder form and were stored in darkness until their addition into the feeding bottles.

For High Performance Liquid Chromatography (HPLC) analysis, milli-Q grade water was produced in house by a Milli-Q purification system (Millipore Corp., Bedford, MA, USA). Acetonitrile and methanol (HPLC solvents) were purchased from Merck (Dramstadt, Germany), and acetic acid from Labbox Labware SL (Madrid, Spain). For peak identification, the following reference substances were used: the HPLC grade 3,4-DHBA (protocatechuic acid) (> 97%), 3,4-DHPE (hydroxytyrosol, > 98%), 4-HPE (tyrosol, 98%), 3,4-DHPE-EA-glucoside (oleuropein, > 98%), and verbascoside (> 99%) were from Merck (Dramstadt, Germany). The 3,4-DHPE-DA-EDA (oleacein), quercetin-3-*O*-rutinoside (rutin), quercetin-3-*O*-glucoside, apigenin-7-*O*-rutinoside, luteolin and apigenin were from Extrasynthèse (Genay, France).

### Chemical characterization of OLE

#### Analysis of total flavonoid content by HPLC–DAD

Determination of total flavonoids as luteolin equivalents was carried out according to Talhaoui et al.^23^.

#### Identification of phenolic compounds by RP-HPLC-PAD-MS(ESI)

Solutions of 10 mg/mL of OLE were prepared in water (in duplicate) and were analysed quantitatively by reversed phase high performance liquid chromatography coupled to photo-diode array detector and mass spectrometry detector with electrospray ionization source (RP-HPLC-PAD-MS(ESI)) as described in Silván et al.^74^. Briefly, the chromatographic system was an Agilent 1100 series (binary pump, autosampler, photodiode array detector (PAD) (Palo Alto, CA, USA)), coupled to an ESI source and quadrupole mass analyser. Stationary phase was an ACE-C18-AR (200 mm × 4.6 mm, 3 μm particle size) column from Advanced Chromatography Technologies (Aberdeen, UK). The mobile phase was a linear gradient of eluent A (2% (v/v) acetic acid in water), and eluent B (2% (v/v) acetic acid in acetonitrile) and was pumped at a flow rate of 0.6 mL/min as follows: from 0 to 80 min, 0% to 20% of B; from 80 to 115 min, 20% to 29% of B; from 115 to 120 min, 29% to 100% of B; during 10 min, 100% of B; and from 130 to 140 min, 100% to 0% of B. The PAD was set at 240, 280 and 330 nm and the injection volume was 10 µL. ESI operation parameters were fixed at: nebulizing pressure of 40 psi and a capillary tension of 4000 V. Drying gas was N_2_, at a flow of 10 L/min at 340 °C. Mass spectra were registered by scanning negative ions from m/z less than 200 Da, at 100 V, m/z 200–1000 Da, at 200 V, and m/z 1000–2500 Da, at 250 V^74^.

### In vivo study

#### Animals

Three (Young; n = 11) and 24-months-old (Old; n = 14) male Wistar rats were fed ad libitum with a standard chow (322.6 kcal/100 g) and housed in climate-controlled quarters with a 12 h light cycle and under controlled conditions of humidity (50–60%) and temperature (22–24 °C). All the experiments and handling of animals were conducted with the approval of the Animal Care and Use Committee of the Community of Madrid (Spain) (PROEX 048-18) according to the European Union Legislation and in compliance with the ARRIVE guidelines.

#### Treatment

Three weeks before sacrifice half of the old rats (Old + OLE) were treated daily with 100 mg/kg of the OLE by dissolved in the drinking water. The other half of the old rats and the young rats received just tap water. Rats were allowed to drink ad libitum. To ensure the correct OLE dose (100 mg/kg/day), the amount of OLE that was added to the drinking water was adjusted every three days depending on both water intake and body weight.

As described at González-Hedström et al.^67^, a daily control of body weight was performed over the three-week treatment. Each day, the change in body weight was calculated by subtracting the daily body weight of each animal from the their body weight at the beginning of treatment. Food intake was assessed weekly by placing a specific amount of chow in each cage and measuring the remaining amount one week later. The day of sacrifice, all animals were injected an overdose of sodium pentobarbital (100 mg/kg) and killed by decapitation after overnight fasting. Before sacrifice, glycemia was measured by venous tail puncture using Glucocard G (Arkray Factory, Inc., Koji Konan-cho, Koka, Shiga, Japan).

To obtain the serum after sacrifice, trunk blood was collected and centrifuged at 3000 rpm for 20 min. After that, visceral (epididymal), subcutaneous (lumbar), brown (interscapular) and perivascular (aortic) adipose tissue depots as well as kidneys, adrenal glands, spleen, liver and heart were immediately removed, weighed and stored at -80 °C for further analysis.

#### Serum measurements

Lipid profile and serum levels of metabolic hormones and inflammatory markers were measured as previously described in González-Hedström et al.^67^.

##### Metabolic hormones

The serum concentrations of leptin, insulin and adiponectin were measured by ELISA kits (Merck Millipore, Dramstadt, Germany) following the manufacturer’s instructions. The sensitivity of the method for leptin, insulin and adiponectin was 0.04, 0.2, and 0.16 ng/mL respectively. The intraassay variation was between 1.9–2.5% for leptin, 0.9–8.4% for insulin, and 0.43–1.96% for adiponectin.

##### Lipid profile

Triglycerides, total lipids, total cholesterol, low-density lipoprotein (LDL), and high-density lipoprotein (HDL) were measured in the serum using commercial kits from Spin React S.A.U (Sant Esteve de Bas, Gerona, Spain) following the manufacturer’s instructions.

### Pro-inflammatory mediators

Interleukin-6 (IL-6) and tumor necrosis factor alpha (TNF-α) plasma levels were measured by an ELISA kit (Cusabio, Wuhan, China) following the manufacturer’s instructions. The sensitivity of the method was 0.078 pg/mL for IL-6 and 1.56 pg/mL for TNF-α. The intraassay and interassay variations were < 8% and < 10% respectively for both.

#### Incubation of aorta segments in presence/absence of insulin (10^−7^ M)

As previously described^67^, 2 mm-long segments from the thoracic aorta were placed in 6 well culture plates and incubated with 1 mL of Dulbecco’s Modified Eagle’s Medium and Ham’s F-12 medium (DMEM/F-12) with glutamine from Gibco (1:1 mix; Invitrogen, Carlsbad, CA, USA), supplemented with 100 U/mL penicillin and 100 μg/mL streptomycin (Invitrogen, Carlsbad, CA, USA) in the presence/absence of insulin (10^−7^ M) (Sigma-Aldrich, St. Louis, MO, USA) at 37 °C in a 95% O_2_ and 5% CO_2_ incubator. The segments and the culture media were collected and stored at − 80 °C for further analysis after 30 min of incubation.

#### Nitrite and nitrate concentrations in the culture medium

As in González-Hedström et al.^67^, using a modified method of the Griess assay^75^, nitrite and nitrate concentrations were measured in the culture medium from aorta segment incubations. Briefly, to 100 μL of culture medium on a 96-well plate, 100 μL of vanadium chloride (Sigma‐Aldrich, St. Louis, MO, USA) were added. Immediately after, 100 μL of the Griess reagent (1:1 mixture of 1% sulfanilamide (Merck Millipore, Darmstadt, Germany) and 0.1% naphthylethylenediamine dihydrochloride (Merck Millipore, Darmstadt, Germany)) were added to each well and incubated at 37 °C for 30 min. Absorbance was measured at 540 nm after incubation. A NaNO_2_ standard curve was used to calculate nitrite and nitrate concentrations. Results were expressed in μM.

#### Protein quantification by Western Blot

An amount of 100 mg of arterial tissue was homogenized using RIPA buffer. After centrifugation (12.500 rpm; 4^○^C, 20 min), the supernatant was collected, and total protein content was analyzed by the Bradford method^76^. As previously described^67^, for each assay, resolving gels with SDS acrylamide (10%) (Bio-Rad, Hercules, CA, USA) were used and 100 μg of protein were loaded in each well. After electrophoresis, proteins were transferred to polyvinylidine difluoride (PVDF) membranes (Bio-Rad, Hercules, CA, USA) and transfer efficiency was determined by Ponceau red dyeing (Sigma-Aldrich, St. Louis, MO, USA). Membranes were then blocked with Tris-buffered saline (TBS) containing 5% (w/v) non-fat dried milk for non-phosphorylated protein or with 5% (w/v) bovine serum albumin (BSA) for phosphorylated protein and incubated with the appropriate primary antibody for Akt (1:1000; Merck Millipore; Dramstadt, Germany) and p-Akt (Ser^473^) (1:500; Cell Signaling Technology; Danvers, MA, USA). Membranes were subsequently washed and incubated with the corresponding secondary antibody conjugated with peroxidase (1:2000; Pierce, Rockford, IL, USA). Peroxidase activity was visualized by chemiluminescence and quantified by densitometry using BioRad Molecular Imager ChemiDoc XRS System (Hércules, CA, USA). All data are referred to % of control values (samples from young rats) on each gel.

#### Experiments of vascular reactivity

After sacrifice, the aorta was dissected and cut into 2 mm long segments for performing experiments of vascular reactivity as previously described^67^. Each segment was prepared for isometric tension recording in a 4-mL organ bath containing modified Krebs–Henseleit solution at 37 °C (mM): NaCl, 115; KCl, 4.6; KH_2_PO_4_, 1.2; MgSO_4_, 1.2; CaCl_2_, 2.5; NaHCO_3_, 25; glucose, 11. The solution was equilibrated with 95% O_2_ and 5% CO_2_ to a pH of 7.3–7.4. Briefly, two fine steel wires (100 µm) were passed through the lumen of the vascular segment. One wire was fixed to the organ bath wall while the other was connected to a strain gauge for isometric tension recording (Universal Transducing Cell UC3 and Statham Microscale Accessory UL5, Statham Instruments, Inc.). This arrangement allows to apply passive tension in a perpendicular plane to the long axis of the vascular cylinder. The changes in isometric force were recorded using a PowerLab data acquisition system (AD Instruments, Colorado Springs, CO, USA). After the setting process, an optimal passive tension of 1 g was applied to the vascular segments. After 60–90 min of equilibration, the vascular segments were stimulated with potassium chloride (100 mM) to determine the contractility of smooth muscle. Segments that failed to contract at least 0.5 g to KCl were discarded.

Using abdominal aortic segments, the vasoconstrictor response to accumulative doses of noradrenaline (10^−9^–10^−4^ M), endothelin-1 (ET-1) (10^−9^–10^−7^ M) or angiotensin-II (AngII) (10^−11^–10^−6^ M) was recorded. Results were expressed as percentage of the contraction to 100 mM KCl.

Thoracic aortic segments were used to study the vasodilator response. They were precontracted with phenylephrine 10^−7.5^ M to subsequently perform a cumulative dose–response curve in response to acetylcholine (10^−9^–10^−4^ M), sodium-nitroprusside (10^−9^–10^−5^ M) or insulin (10^−8^–10^−5^ M). Relaxation was expressed as percentage of the initial tone.

All drugs were obtained from Sigma-Aldrich, (St. Louis, MO, USA).

#### RNA extraction and purification

As in González-Hedström et al.^67^, total RNA was extracted from myocardial, liver and arterial tissue according to the Tri-Reagent protocol^77^ and quantified with Nanodrop 2000 (Thermo Fisher Scientific, Hampton, NH, USA). From 1 µg of total RNA, cDNA was synthesized by a high capacity cDNA reverse transcription kit (Applied Biosystems, Foster City, CA, USA)^67^, total RNA was extracted from myocardial, liver and arterial tissue according to the Tri-Reagent protocol^77^ and quantified with Nanodrop 2000 (Thermo Fisher Scientific, Hampton, NH, USA). From 1 µg of total RNA, cDNA was synthesized by a high capacity cDNA reverse transcription kit (Applied Biosystems, Foster City, CA, USA).

#### Quantitative real-time PCR

The gene expression of interleukin 1β (IL-1β, Rn00580432_m1), interleukin 6 (IL-6, Rn01489669_m1), interleukin 10 (IL-10, Rn01483988_g1), tumoral necrosis factor alpha (TNF-α, Rn01525859_g1), cyclooxigenase-2 (COX-2, Rn01483828_m1), inducible Nitric Oxide Synthase (iNOS, Rn00561646_m1), NADPH oxidase 1 (NOX1, n00586652_m1), NADPH oxidase 4 (NOX4, Rn00585380_m1), glutathione reductase (GSR, Rn01482159_m1), glutathione peroxidase (GPx, Rn00574703_m1), glucokinase (GCK, Rn00561265_m1), glycogen synthase kinase 3β (GSK3β, Rn01444108_m1), superoxide dismutase-1 (SOD1, Rn00566938_m1) and lipoxygenase (Alox5, Rn00563172_m1) were assessed in myocardial, liver and arterial tissues by quantitative real-time polymerase chain reaction (qPCR) using assay-on-demand kits (Applied Biosystems, Foster City, CA, USA) as previously described^67^. TaqMan Universal PCR Master Mix (Applied Biosystems, Foster City, CA, USA) was used for amplification according to the manufacturer’s protocol in a Step One machine (Applied Biosystems, Foster City, CA, USA). Values were normalized to the housekeeping gene 18S (Rn01428915_g1). According to manufacturer’s guidelines, the ∆∆C_T_ method was used to determine relative expression levels^78^.

#### Statistical analysis

Values are expressed as means ± standard error of the mean (SEM) and analyzed by one-way ANOVA followed by Bonferroni post-hoc test using GraphPad Prism 5.0. (San Diego, California, USA). Body weight gain over time was analyzed by two-way ANOVA. A *p* value of < 0.05 was considered significant.

## Supplementary Information

Supplementary Information

## Data Availability

All data generated or analyzed during this study are included in this published article.
