# Supplementary material for: Olive leaf extract supplementation improves the vascular and metabolic alterations associated with aging in Wistar rats
Source: Sci Rep. 2021 Apr 14;11:8188. doi: 10.1038/s41598-021-87628-7 (PMC8046982; doi:10.1038/s41598-021-87628-7)
Supplement: Supplementary file 1 — Supplementary Information [file 41598_2021_87628_MOESM1_ESM.docx]

**Olive leaf extract supplementation improves the vascular and metabolic alterations associated with aging in Wistar rats**

González-Hedström D^1,2^, García-Villalón AL^1^, Amor S^1^, de la Fuente-Fernández M^1^, Almodóvar P^2^, Prodanov M^3^, Priego T^,4^, Martín AI^,4^, Inarejos-García AM^2^, Granado M^1,5*^

^1^ Departamento de Fisiología, Facultad de Medicina, Universidad Autónoma de Madrid, Madrid, Spain.

^2^ Pharmactive Biotech Products S.L. Parque Científico de Madrid. Avenida del Doctor Severo Ochoa, 37 Local 4J, 28108 Alcobendas, Madrid

^3^ Departamento de Química Física Aplicada, Facultad de Ciencias, CIAL (CEI, CSIC-UAM), Universidad Autónoma de Madrid, Madrid, Spain

^4^ Departamento de Fisiología, Facultad de Medicina, Universidad Complutense de Madrid, Madrid, Spain.

^5^ CIBER Fisiopatología de la Obesidad y Nutrición. Instituto de Salud Carlos III, Madrid.

* Correspondence: miriam.granado@uam.es; Tel.: +34‐914‐976‐974

***SUPPLEMENTARY INFORMATION***

**A**


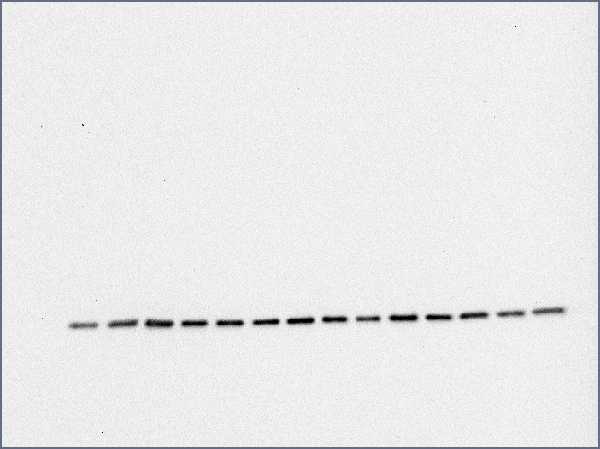

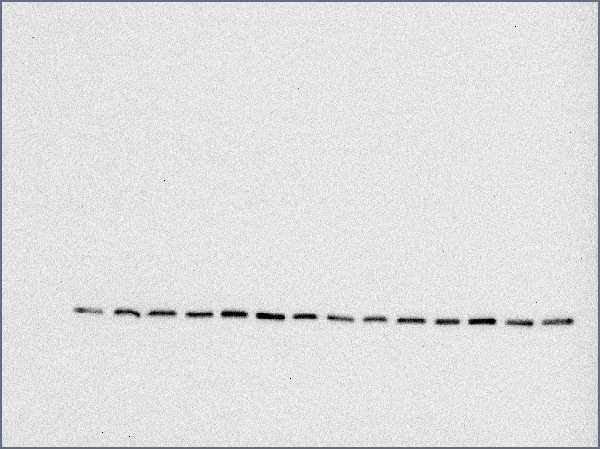

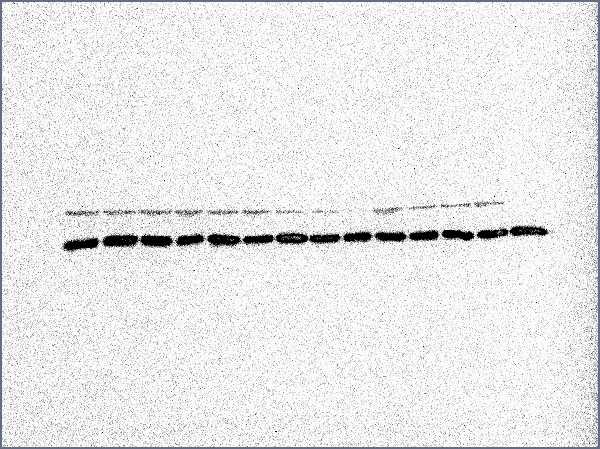

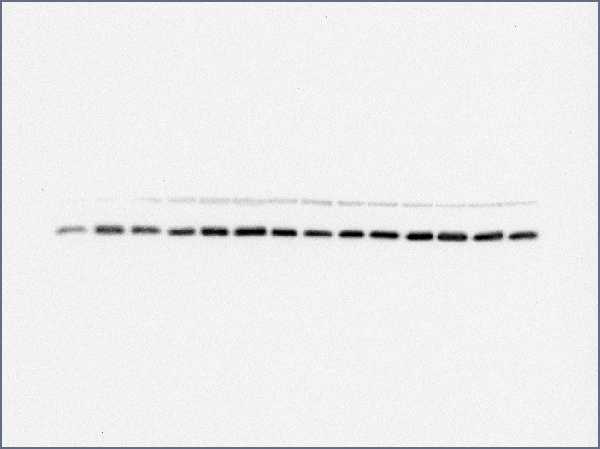


Young

Old

Old + OLE

C

I

C

I

C

I

**B**

Young

Old

Old + OLE

C

I

C

I

C

I

**Supplementary Figure 1**. Original and unprocessed blots of p-Akt western blot (**A**) analysis from Figure 6C and their control blots of GAPDH (**B**). *Showed blots at Figure 6C are marked with red boxes.* *C = Control; I = Insulin; OLE = olive leaf extract.*


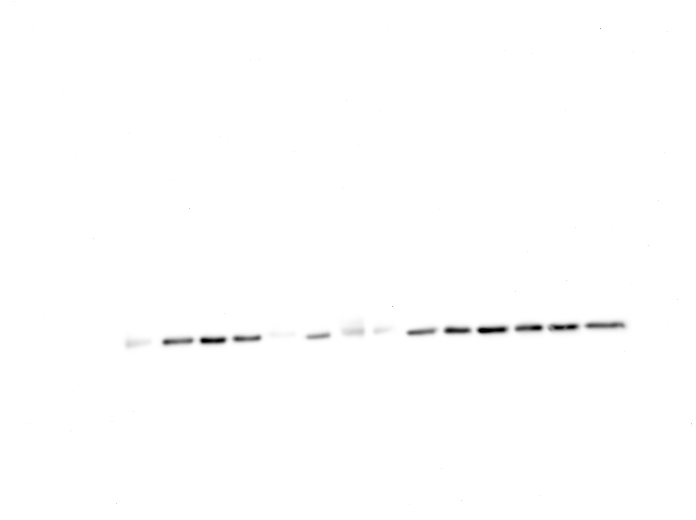

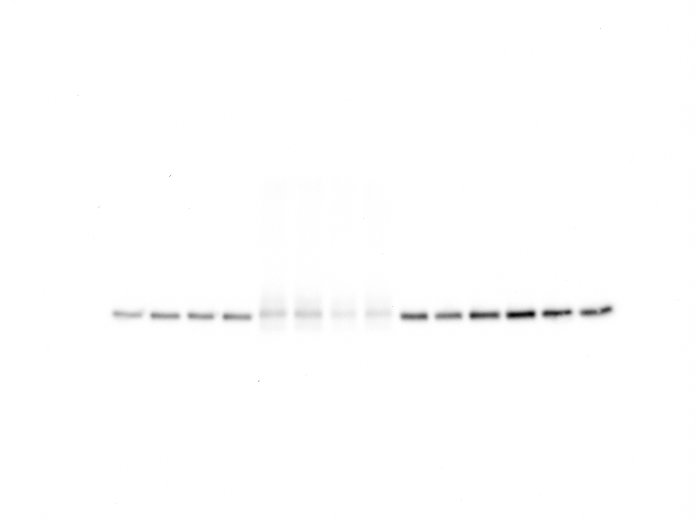


**B**

**A**

Old

Young

Old + OLE

C

I

C

I

C

I


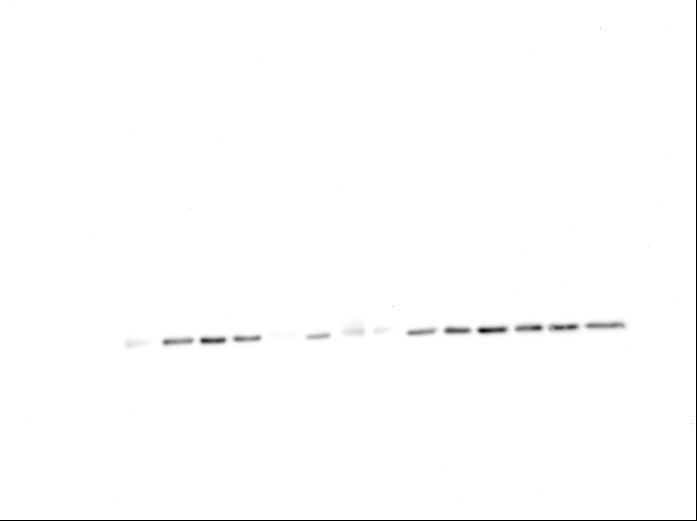

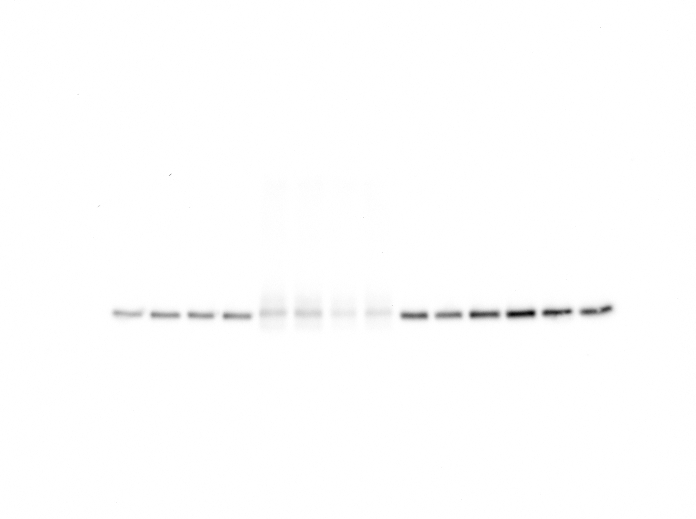


Young

Old

Old + OLE

C

I

C

I

C

I

**C**


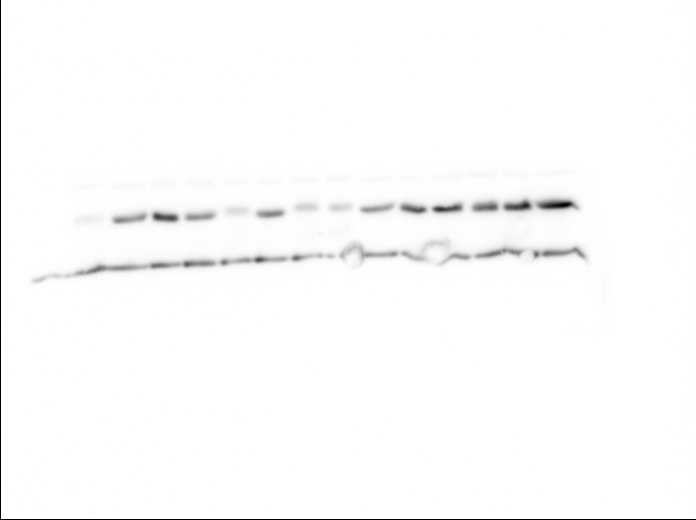

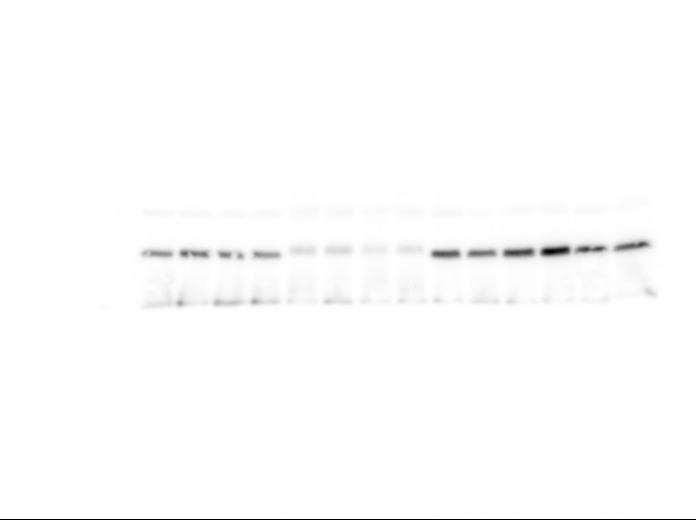


Young

Old

Old + OLE

I

C

I

C

I

C

**D**


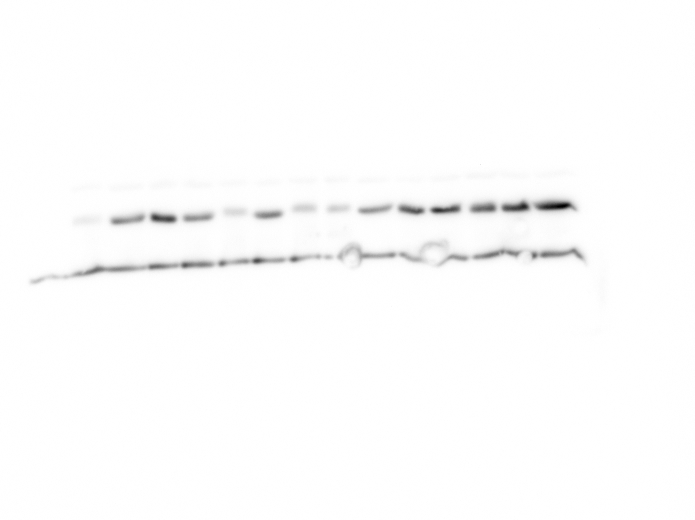

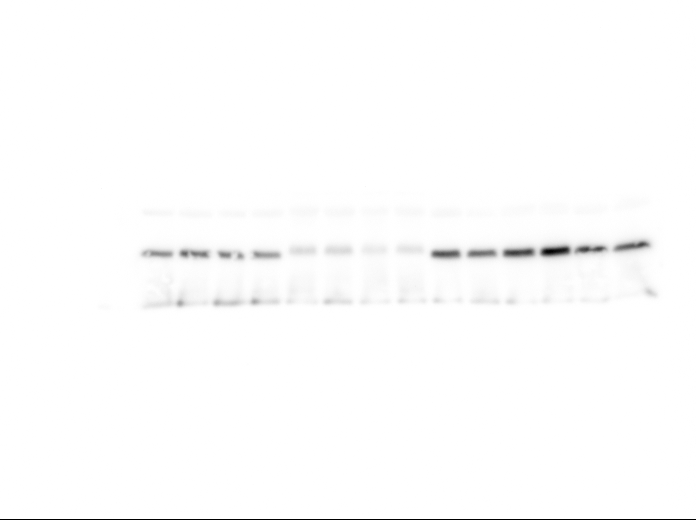


Young

Old

Old + OLE

I

C

I

C

I

C

**Supplementary Figure 2.** Original and unprocessed blots of Akt western blot analysis from Figure 6C with 9 (**A**) and 6 (**B**) seconds of exposure, and their control blots of GAPDH with 5 (**C**) and 12 (**D**) seconds of exposure. *Showed blots at Figure 6C are marked with red boxes.* *C = Control; I = Insulin; OLE = olive leaf extract.*

**Supplementary Figure 3.** Effects of aging and a 21-day treatment with the OLE on the ratio between protein levels of p-Akt and Akt (**A**) and on the ratio between protein levels of p-GSK3β and GSK3β (**B**) in the liver. Values are represented as mean ± SEM. * *p* < 0.05 vs. Young.


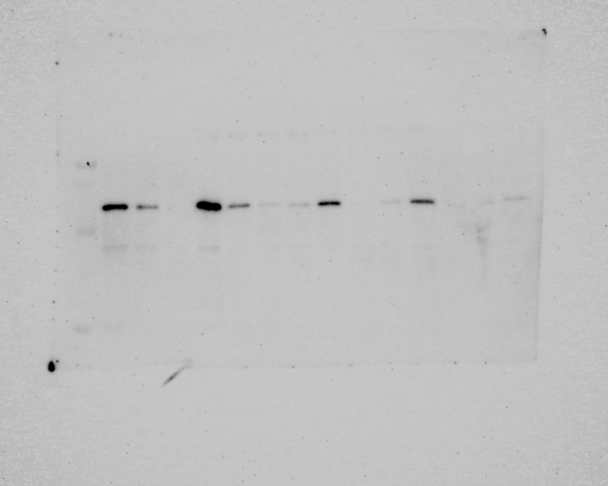

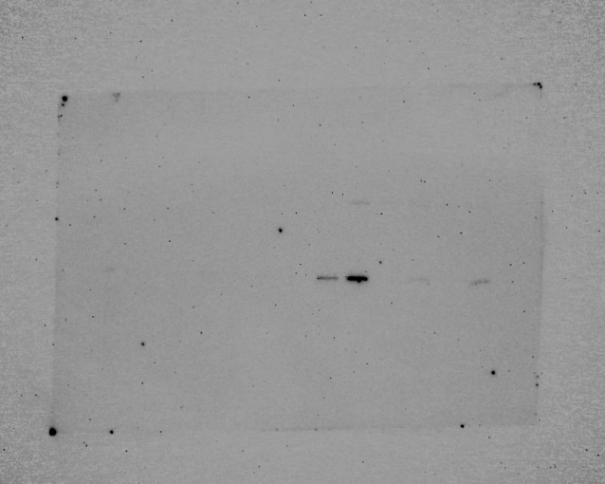


O

Ole

Y

**B**

**A**


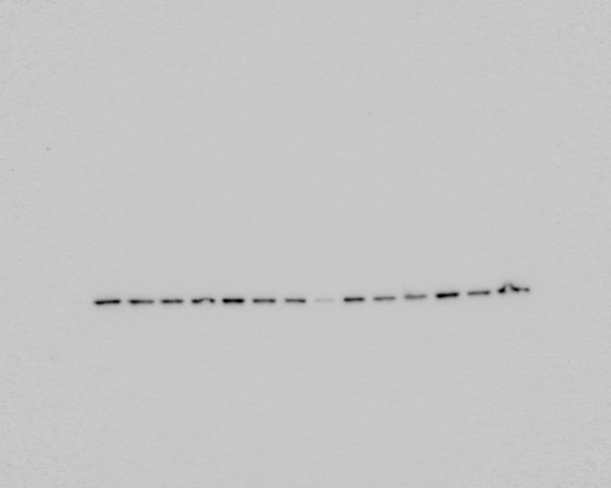

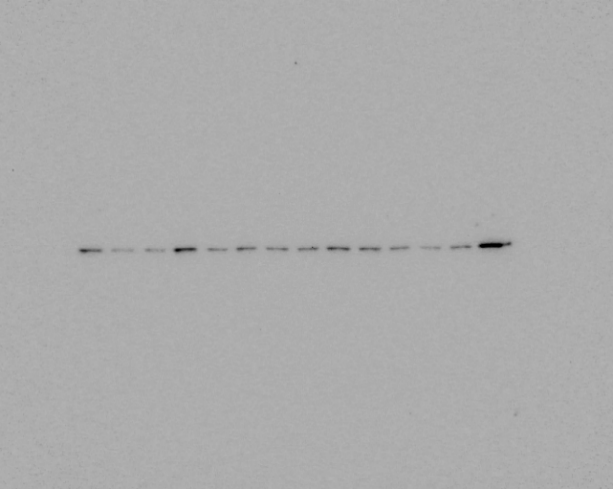


Ole

O

Y

**C**


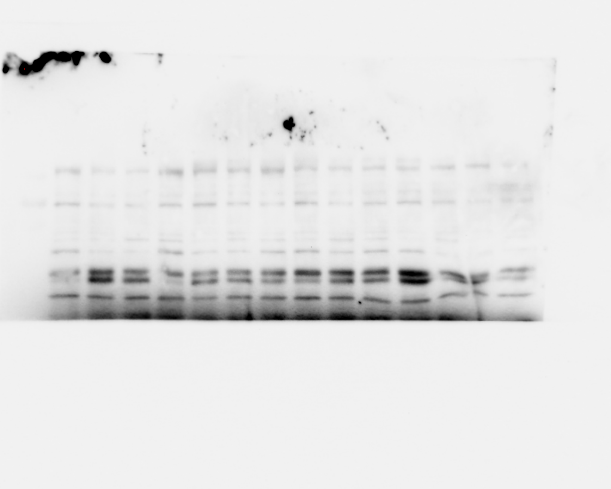

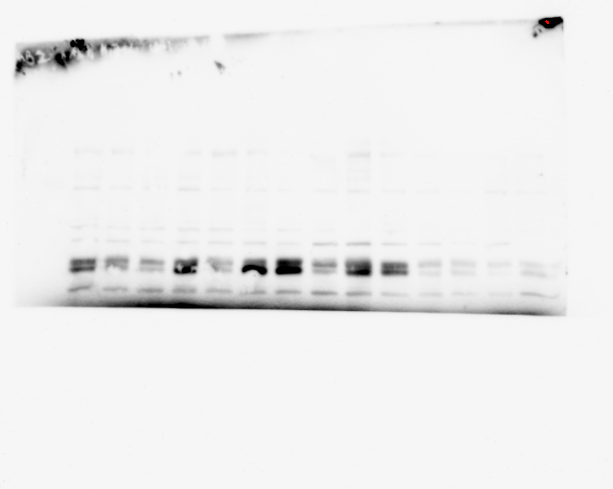


O

Ole

Y

**D**


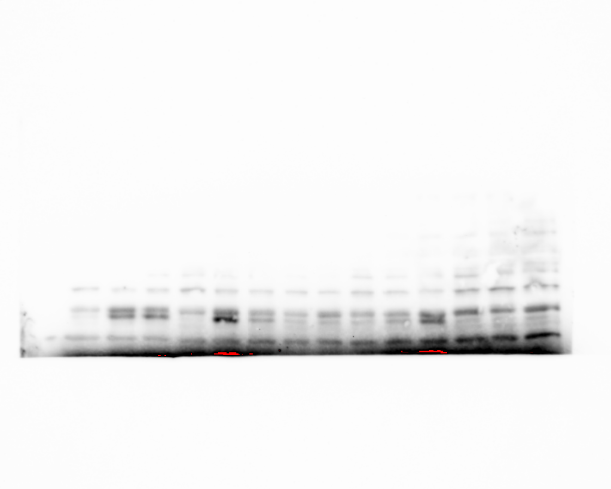

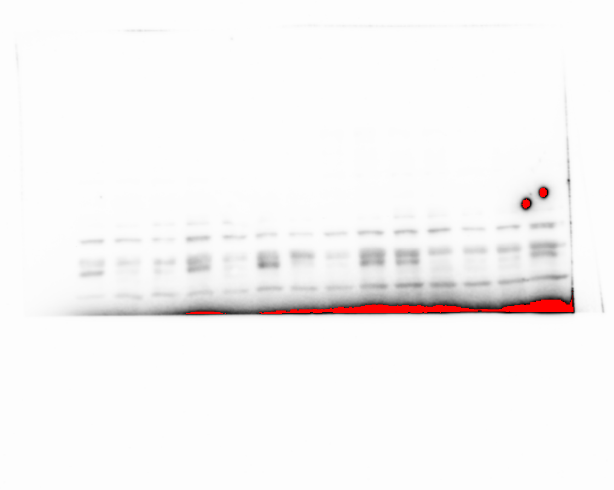


O

Ole

Y

**Supplementary Figure 4**. Original and unprocessed blots of p-Akt (**A**), total Akt (**B**), p-GSK3β (**C**), and total GSK3β (**D**) western blot analysis from Supplementary Figure 3. *Showed blots at Supplementary Figure 3 are marked with red boxes.* *Y = Young; O = Old; Ole = Old + olive leaf extract.*
